# Understanding drought response mechanisms in wheat and multi-trait selection

**DOI:** 10.1371/journal.pone.0266368

**Published:** 2022-04-14

**Authors:** Maicon Nardino, Ellen Cristina Perin, Bianca Camargo Aranha, Solange Teresinha Carpes, Bruno Henrique Fontoura, Diana Jhulia Palheta de Sousa, Davi Soares de Freitas

**Affiliations:** 1 Department of Agronomy, Federal University of Viçosa, Viçosa, MG, Brazil; 2 Department of Chemistry, Postgraduate Program in Chemical and Biochemical Process Technology, Federal Technological University of Paraná campus Pato Branco, Pato Branco, PR, Brazil; 3 Department of Crop Protection, Federal University of Pelotas, Pelotas, RS, Brazil; Institute of Genetics and Developmental Biology Chinese Academy of Sciences, CHINA

## Abstract

Wheat crop is very sensitive to osmotic stress conditions. As an abiotic stress, drought may exert a considerable effect on the levels of specialized metabolites in plants. These metabolites may exert beneficial biological activities in the prevention or treatment of disorders linked to oxidative stress in plants and humans. Furthermore, osmoprotector accumulation helps wheat to increase the maintenance of osmotic balance. Therefore, identifying wheat genotypes with better drought tolerance is extremely important. In this sense, this research aimed to understand agronomic, physiological and biochemical responses of spring wheat strains and cultivars to drought stress, under field conditions, and jointly select strains via multi-trait index. We evaluated agronomic, physiological and biochemical variables in 18 genotypes under field condition. The results demonstrated that all variables were affected by the drought. Most genotypes were significantly reduced in grain yield, except VI_14774, VI_14668, VI_9007 and TBIO_ATON. The variables related to photosynthesis were also affected. An increase above 800% was observed in proline contents in genotypes under drought. Sodium and potassium also increased, mainly for VI_131313 (Na), while VI_130758 and VI_14774 presented increased K. We evaluated the antioxidant potential of the different strains and the total content of phenolic compounds. The most drought-responsive genotypes were BRS_264, VI_14050 and VI_14426. Reduced grain yield and photosynthetic variables, and increased specialized metabolism compounds are due to plant defense mechanisms against drought conditions. Furthermore, variation in genotypes can be explained by the fact that each plant presents a different defense and tolerance mechanism, which may also occur between genotypes of the same species. Four strains were selected by the multivariate index: VI_14055, VI_14001, VI_14426 and VI_1466. Such results allow us to predict which genotype(s) performed best in semi-arid environments and under climatic fluctuations.

## 1. Introduction

Wheat (*Triticum aestivum* L.) is one of the three most consumed cereal crops in the world, along with rice and corn, and a relevant source of nutrients for the population [1,2]. While primarily an energy supplier, wheat is also rich in fiber, protein, vitamins and minerals [3]. World wheat production in the 2019/2020 marketing year surpassed 765 million tons, which means an increase of more than 30 million tons compared to the previous year. Furthermore, a production of approximately 776 million tons is expected for 2021 [4]. The largest wheat producing countries were those of the European Union, China and India [5]. Brazil is ranked in the sixteenth position in wheat production [6].

It is estimated that food production will have to increase significantly to keep up with the projected population demand for 2050 with approximately 9 billion people, as predicted by the [7]. The greater demand in food production, linked to the climate change phenomenon observed in recent years, suggests the need for greater productivity. In order to make wheat production gains to accompany population increase, the genotypes must be more productive and new arable areas must be explored, including many with limiting conditions for the production of the wheat crop [8].

Climate change is increasingly affecting agriculture and the agronomic performance of cultivated species. Drought is one of the most important limiting phenomena in wheat production and yield, since drought stress causes greater losses to grain yield when it coincides with the reproductive period [9]. However, plants have morphological, biochemical, physiological and molecular mechanisms of response to drought stress.

Under osmotic stress conditions, plant metabolism is affected, the photosynthetic machinery function is compromised, and the leaves start to enter into senescence. These factors occur due to the oxidative stress caused by the accumulation of reactive species that damage cell structure and function in chloroplasts [10]. In wheat, the flag leaf is the main photosynthetic and energy-producing structure, which is essential for the crop to complete its cycle. The commitment of the flag leaf induces grain productivity loss [11]. Oxidative stress can be countered through the accumulation of non-enzymatic antioxidant compounds (phenolics and carotenoids) and antioxidant enzyme complexes [12,13].

In wheat, as in other plants, antioxidant metabolites are constantly produced in an attempt to maintain the homeostatic cells. However, the production and variation of the compounds depend on the conditions of cultivation, development and the defense against biotic and/or abiotic stresses that may occur [12,14]. The main compounds related to the antioxidant defense in wheat belong to the class of secondary metabolism, hereinafter called specialized, mainly in the case of wheat produced phenolic compounds and terpenes, in addition to enzymatic antioxidant substances [15,16]. These compounds have antioxidant potential, due to the mechanisms of action involved, in neutralizing, sequestering and/or donating electrons to reactive and unstable substances produced, the free radicals [17].

Studies addressing biotic and abiotic stresses in wheat corroborate the presence of antioxidant substances under such situations and reveal variations in the amount and profile of compounds, according to the condition and genotype involved, both in grains and leaves, as well as under conditions of water deficit and high nitrogen treatments [18], in resistance towards aphid complex [19] salt stress [20], during grain development [21], UV-B radiation [22], genotype and stress by temperature [15], among other studies.

In addition to the effect of stress on specialized metabolism, dehydration increases the control of stomatal opening and closure in order to reduce water losses during evapotranspiration, consequently reducing stomatal conductance, CO_2_ ingress and the rates of liquid photosynthesis [14,23]. Another important factor is the compromised osmotic adjustment that causes turgor loss and osmotic imbalance. Therefore, wheat osmotic potential must be reduced to maintain cell function during dehydration [13].

The accumulation of osmoprotectors, also known as compatible osmolytes or solutes, polar and uncharged, such as proline, glycinebetaine, sugar alcohols and ions, helps wheat to perform basic metabolic functions and mainly improves the maintenance of osmotic balance, the protection of organelles and cells facing dehydration, stabilization of membranes and structures of proteins and enzymes, and detoxification of ROS [24,25].

Three mechanisms can be categorized to trigger these responses: prevention, escape or drought tolerance. Regarding prevention, it can be characterized by increased maintenance of the water potential, even under conditions of low soil moisture content or by increasing the amount of water absorption. As for the escape, it induces plant precocity, without going through the plant terminal stress. Finally, tolerance, the focus of several studies, mainly in the area of genetic breeding, is due to the maintenance of turgor by osmotic adjustment, which increases cell elasticity and reduces its size. Furthermore, in these cases, the plant produces equal or even greater economic income [26,27].

However, few studies seek to understand the behavior of different wheat genotypes under water deficit regarding biochemical-physiological mechanisms and agronomic aspects. Most studies are carried out under controlled conditions in greenhouses. However, the selection of strains that tolerate such drought conditions is a strategic alternative to contribute not only to the issue of water resources, but also to the continued high grains production and maintenance of technological quality.

Multivariate data information is common in biological experiments, and using multiple traits is crucial to make better decisions for genotype selection. However, identifying genotypes or treatments that combine high performance across many traits has been a challenging task. Due to the main classical indices, it has a problem with the presence of multicollinearity and the arbitrary choice of weighting coefficients. Thus, a recent proposal named multi-trait genotype-ideotype distance index (MGIDI), based on a mixed model, provides a multivariate selection process free from multicollinearity and weighting coefficients [28].

Combining information from the areas of agronomic, biochemical and genetic breeding research, in addition to the use of different statistical tools, may increase knowledge about tolerance drought and help to identify promising wheat genotypes, under drought conditions. In this sense, this research aimed to understand agronomic, physiological and biochemical responses of spring wheat strains and cultivars to drought stress, under field conditions, and jointly select strains via multi-trait index.

## 2. Material and methods

### 2.1 Field experiments

Field experiments were carried out between May 2020 and February 2021, in the experimental area of the Department of Agronomy of the Federal University of Viçosa–UFV, located in Viçosa-MG (20°45’14" S; 42°52’55" W, a 648 m de altitude). The biochemical determinations were conducted in the chemistry laboratories of the Federal Technological University of Paraná, Pato Branco Campus.

The genotypes were arranged in a completely randomized block design with three replications, in a factorial scheme (control and drought). Eighteen wheat genotypes were used, including two important commercial cultivars, which are the most frequently used by farmers. Sixteen wheat lines were developed by our UFV public breeding program (Table 1). The experimental plot consisted of 5-five m long cultivar rows spaced at 0.20 m, with a final population density of 350 plants m^−2^.

**Table 1:** Wheat genotypes (two cultivars and 16 strains in a crop value and use (VCU) assay of the UFV Wheat Program) submitted to control condition and drought stress.

ID control	ID drought	Genotype	Company	Cycle
1	19	BRS_264	Embrapa	Early
2	20	TBIO_ATON	Biotrigo Genética	Medium
3	21	VI_130679	UFV	Medium
4	22	VI_130755	UFV	Medium
5	23	VI_130758	UFV	Medium
6	24	VI_131313	UFV	Medium
7	25	VI_14001	UFV	Early
8	26	VI_14026	UFV	Early
9	27	VI_14050	UFV	Early
10	28	VI_14055	UFV	Early
11	29	VI_14118	UFV	Early
12	30	VI_14426	UFV	Early
13	31	VI_14668	UFV	Early
14	32	VI_14774	UFV	Early
15	33	VI_14867	UFV	Early
16	34	VI 14950	UFV	Early
17	35	VI_14980	UFV	Early
18	36	VI_9007	UFV	Early

### 2.2 Management

Basic fertilization was carried out according to the interpretation of the chemical analysis of the soil aiming to meet the crop requirements. In the sowing furrow, 300 kg ha^−1^ of the formula 08-28-16 (nitrogen, phosphorus, potassium) were applied. As cover fertilization, 90 kg ha^−1^ of nitrogen (N) were applied in two phases, 50% at the beginning of tillering and 50% at the beginning of the booting stage. Urea (45% N) was used as the nitrogen source, totaling 200 kg ha^−1^.

### 2.3 Control stress and irrigation

Eighteen genotypes were submitted to irrigation (control samples) and drought stress condition. Two experiments were simultaneously conducted: one experiment was carried out using sprinkler irrigation according to the water needs of the crop. The other was conducted with restricted irrigation at stages of phenological heading [29]. The time of stress was 30 days, which coincided with wheat physiological maturation. The experimental areas were approximately 20 meters apart from each other. Initially, soil samples were collected at depths of 0–10 and 10–20 cm for each environment (S1 Fig). These samples were homogenized and sent to the chemical analysis laboratories to obtain the soil water retention curve. For the monitoring of soil moisture, the samples were taken every two days by soil collection at 10 points of each environment with the aid of the drought, at depths of 0–10 and 10–20 cm. Then, the soil samples were weighed and placed in an oven with air circulation of 60°C, for 48 hours. Later, they were weighed again and the amount of water in the soil was estimated.

The soil physical analysis data for soil water retention curve (CRA, kpa) were: -10kpa = 0.391 kg/kg; -30kpa = 0.35kg/kg; -50kpa = 0.327kg/kg; -100kpa = 0.294kg/kg; -300kpa = 0.274kg/kg; -1500kpa = 0.234kg/kg.

### 2.4 Data collection

We evaluated the following agronomic traits: grain yield (GY, kg ha^-1^), hectolitre weight (HLW, kg hL^−1^); physiological variables: liquid photosynthesis initial and final (AI and AF μmol de CO_2_ m^- 2^ s^-1^), stomatal conductance initial and final (gsI and gsF, mol H_2_O m^-2^ s^-1^), proline content (pc, μg g^-1^), sodium (Na, mg g^-1^) and potassium (K, mg g^-1^); and the biochemical traits: total phenolic compounds (TPC, mg GAE g^-1^) and antioxidant activity by 2,2 -azino-bis (3-ethylbenzothiazoline-6-sulfonic acid) (ABTS, mM TEAC g^-1^) and ferric reducing antioxidant power (FRAP, mM Fe^+2^ g^-1^) methods, according to the methodologies described below.

#### 2.4.1 Agronomic and physiological traits measured

GY was determined by manually harvesting the five central cropping rows, adjusting to 13% moisture and converting the grain weight to the hectare scale; HLW, determined by weighing a known volume (250 mL) of a sample, using a Dalle-Molle scale, and transforming the result into the standard unit.

Gaseous exchange traits were measured in both experiments (stress and control), in two phases. The first assessment was conducted at the beginning of stress, and the last, about 25 days later. The assessment of gas exchange was performed at the time of crop anthesis and milky grain (phase 65 and 75), according to the phenological scale of [29], in the morning, without cloudiness and without wetting in the canopy, on the flag leaf of a plant in the central row. For this purpose, an infrared gas analyzer (IRGA) (ACD, LCPro SD, Hoddesdon, UK) was used with an air of 300 mL min^-1^ and 1200 μmol m^-2^ s^- 1^ of the light source, obtaining as a response the A expressed in μmol de CO_2_ m^- 2^ s^-1^ and gs in mol H_2_O m^-2^ s^-1^.

For the pc analysis, the method attributed by [30], was adapted [31] in approximately 0.1 g leaf samples The toluene layer containing chromophore was separated and kept at room temperature for a few minutes, and the absorbance was read at 520 nm in a spectrophotometer, using toluene as blank. The proline concentration was estimated using an L-proline standard curve prepared from L-proline standard (0–25 μg mL^-1^), and the data were expressed in μg of proline g leaf^-1^.

Sodium and Potassium content was assessed according to [32] and [33], and the results were expressed in mg g^-1^.

#### 2.4.2 Biochemical traits measured

All the biochemical analyses were performed in triplicate.

For the quantification of TPC and antioxidant activity by the FRAP and ABTS methods, an extract was obtained according to [34], with adaptations. Wheat leaves (0.1 g previously lyophilized) were added to falcon tubes, using 90% ethanol as extracting agent (10 mL). The mixture was homogenized by vortexing and left in water bath, at 60°C, for 30 minutes. Next, the samples were centrifuged at 10000 rpm, for 10 minutes. The supernatant was collected and stored in a freezer until analysis was performed.

The TPC of the extracts from wheat leaf was determined using the Folin–Ciocalteu method, as described by [35]. The absorbance of the extract was measured at 740 nm in a spectrophotometer (UV–Vis Bel Photonics, 2000 Piracicaba, Brazil) and expressed in mg GAE g^-1^ (GAE: gallic acid equivalent).

The antioxidant activity by the ABTS^+^ method was performed according to the methodology described by [36]. The absorbance was measured at 734 nm, and the results were expressed in mM TEAC g^-1^ sample (TEAC: antioxidant capacity equivalent to Trolox).

The FRAP of the extracts were determined by the procedure described by [37], based on the ability of the antioxidant to reduce Fe^+3^ to Fe^+2^, in the presence of 2,4,6-tri (2 -pyridyl) 1,3,5-triazine (TPTZ). The absorbance readings were performed on a spectrophotometer at 595 nm. The results were expressed in mM Fe^+2^ g^-1^.

### 2.5 Statistical analyses

Each trait was analyzed according to the following mixed-effect model:

y=Xβ+Zu+e

Where **y** is a $\eta [=\sum_{j=1}^{e}(gb)]\times 1$ vector of response trait $y={[y}_{111},y_{111},\ldots y_{geb}]´$, where *g*, *e*, and *b* are the number of genotypes, environments, and blocks, respectively; ***β*** is an (*eb*) × 1 vector of unknown fixed effects $b=[\gamma_{11},\gamma_{12},\ldots ,\gamma_{eb}]´$; **u** is an *m*[= *g*+*ge*]×1 vector of random effects $u=[\alpha_{1},\alpha_{1},\ldots ,\alpha_{g},{\left(\alpha \tau \right)}_{11},{\left(\alpha \tau \right)}_{12},\ldots ,{\left(\alpha \tau \right)}_{ge}]´;$ **X** is an *n ×*(*eb*) design matrix relating **y** to ***β*; Z** is an *n×m* design relating **y** to **u;** and **e** is an *n ×* 1 vector of random errors $e={[y}_{111},y_{111},\ldots y_{geb}]´$. The significance of the genotype effects and interaction were tested by a likelihood ratio (LRT) test.

The mixed model analysis was performed in the R 4.0.1 software system, using the functions gamem() and get_model_data() of the package metan [38].The principal component analysis was realized for the environment jointly with the package *factoextra*. The inputs for the analyses were the data of Best linear unbiased prediction—BLUP’s genotypes and environment. For the correlation analyses, we used the mean values of BLUP, while the Pearson coefficient was used to obtain the estimates. We used the *corrplot* package [39].

#### 2.5.1 Multi-trait genotype-ideotype distance index (MGIDI)

The multi-trait genotype-ideotype distance index (MGIDI) was used to rank the genotypes based on information of multiple traits, as proposed by [38]. The first step to compute the MGIDI was to rescale the matrix **X** so that all the values have a 0–100 range. The rescaled value for the *j*th trait of the *i*th genotype (*r***X**_***ij***_) was obtained as described:

$$rX_{ij}=\frac{n_{nj}-\varphi_{nj}}{n_{oj}-\varphi_{oj}}\times (\theta_{ij}-n_{oj})+n_{nj}$$

Where *n*_*nj*_ and *φ*_*nj*_ are the new maximum and minimum values for the trait *j* after rescaling, respectively; *n*_*oj*_ and *φ*_*oj*_ are the original maximum and minimum values for the trait *j*, respectively, and *θ*_*ij*_ is the original value for the *j*th trait of the *i*th genotype.

The values for *n*_*nj*_ and *φ*_*nj*_ were determined as follows. For the traits in which lower values are desired (na, gsI and gsF), we used *n*_*nj*_ = 0 and *φ*_*oj*_ = 100. For the traits in which higher values are desired (GY, HLW, TPC, ABTS, FRAP, pc, K, AI and AF), we used *φ*_*oj*_ = 100 and *φ*_*oj*_ = 0. After the rescaling procedure, a two-way table of rescaled values (*r***X**) was obtained. Each column of *r***X** has a 0–100 range that considers the desired sense of selection (increase or decrease) and maintains the correlation structure of the original set of variables.

*2*.*5*.*1*.*1*. *Factor analysis (FA)*. The second step was to compute an exploratory factor analysis to group correlated traits into factors and then estimate the factorial scores for each genotype. The eigenvalues and eigenvectors were obtained from the correlation matrix of the two-way table *r***X**. The initial loadings were obtained considering only factors with eigenvalues higher than one. This analysis was performed according to the following model:

$$F=Z(A^{T}R^{-1}{)}^{T}$$

Where F is a *g × f* matrix with the factorial score; Z is a *g × p* matrix with the rescaled; A is a *p × f matrix of canonical loading*, *and* R is a *p × p* correlation matrix between the indices. Furthermore, *g*, *f* and *p* indicate the number of genotypes, factor retained, and calculated indices, respectively.

*2*.*5*.*2*.*2*. *Ideotype*. Ideotype planning was in the third step of the MGIDI computation. By definition, the ideotype has the highest rescaled value (100) for all analyzed traits. Thus, the ideotype was defined by a [1*× p*] vector **I** such that **I =** [100, 100, …, 100]. The multi-trait genotype-ideotype distance index was estimated in the last step (MGIDI), using the following equation:

$$MGIDI_{i}=\sum_{j=1}^{f}{\left[{\left(y_{ij}-y_{j}\right)}^{2}\right]}^{0.5}$$

where *MGIDI*_*i*_ is the multi-trait genotype-ideotype distance index for the *i*th genotype in the *j*th factor (*i =* 1, 2, …, *g*; *j* = 1, 2, …, *f*), where *g* and *f* are the number of genotypes and factors, respectively; and *y*_*j*_ is the *j*th score of the ideotype. The genotype with the lowest MGIDI is then closer to the ideotype and therefore presents desired values for all the analyzed traits. The proportion of the MGIDI of the *i*th genotype explained by the *j*th factor (*ω*_*ij*_) was computed as described below:

$$\omega_{ij}=\frac{\sqrt{D_{ij}^{2}}}{\sum_{j=1}^{f}\sqrt{D_{ij}^{2}}}$$

where *Dij* is the distance between the *i*th genotype and the ideotype for the *j*th factor. For a given genotype, factors with low contributions suggest that such genotype is close to the ideotype for the traits within that factor. The selection gain in percentage, SG (%), was calculated for each trait, considering a selection proportion of 25%.

## 3. Results and discussion

### 3.1 Deviance and descriptive analysis

The LRT test indicated high significance for the random effects of genotype-environment (G×E) (p < 0.005) for the following traits: GY, TPC, ABTS, FRAP, pc, K and Na. Therefore, genotype effects (G) (p < 0.05) are observed in the traits analyzed: HLW, AF and gsF (Table 2). These results demonstrate that the performance of the genotypes changed according to the environment (control and drought). LRT significance was observed significance for the environment effects between the GY, TPC, FRAP, pc, Na, gsI, gsF (S1 Table) traits.

**Table 2:** - Phenotypic variance, broad-sense and genotype mean basis (h^2^) heritability, accuracy of selection (h), genotype-environment correlation (rge), genotypic (CVg) and residual (CVr) coefficient of variation, and CVg/CVr ratio.

Parameters	h^2^	h	rge	CVg(%)	CVr(%)	CV ratio
GY	0.505	0.710	0.651	7.845	7.414	1.058
HLW	0.782	0.885	0.162	1.984	2.041	0.972
AI	0.289	0.538	0.000	3.464	13.300	0.260
AF	0.615	0.784	0.140	10.296	16.357	0.629
gsI	0.313	0.560	0.000	7.213	26.165	0.276
gsF	0.224	0.474	0.305	5.348	16.003	0.334
pc	0.850	0.922	0.970	56.604	5.876	9.633
Na	0.029	0.171	0.905	1.759	4.583	0.384
K	0.352	0.594	0.981	14.753	3.924	3.760
TPC	0.810	0.900	0.383	6.649	4.660	1.427
ABTS	0.524	0.724	0.825	10.840	6.502	1.667
FRAP	0.296	0.544	0.904	7.867	5.502	1.430

Grain yield (GY), hectolitre weight (HLW), initial liquid photosynthesis (AI) and final liquid photosynthesis (AF), initial stomatal conductance (gsI) and final stomatal conductance (gsF), proline content (pc), sodium (Na), potassium (K), total phenolic compounds (TPC) and antioxidant activity by the ABTS and FRAP methods.

In recent decades, the global climate has changed, which resulted in drastic fluctuations in precipitation patterns and rising temperatures [40]. These oscillations are mainly observed in tropical climate regions. Given the scenario of population increase, the demand for wheat-based food, without opening new area frontiers [41,42], is a significant challenge. In relevant research [41], signs of stagnation in wheat grain production were highlighted, especially in regions with frequent water stress and semi-arid climate [43]. This is an important factor to be studied and explored in research, in the search for genotypes more tolerant to such conditions, in an attempt to reduce losses caused by drought conditions.

**Fig 1:**
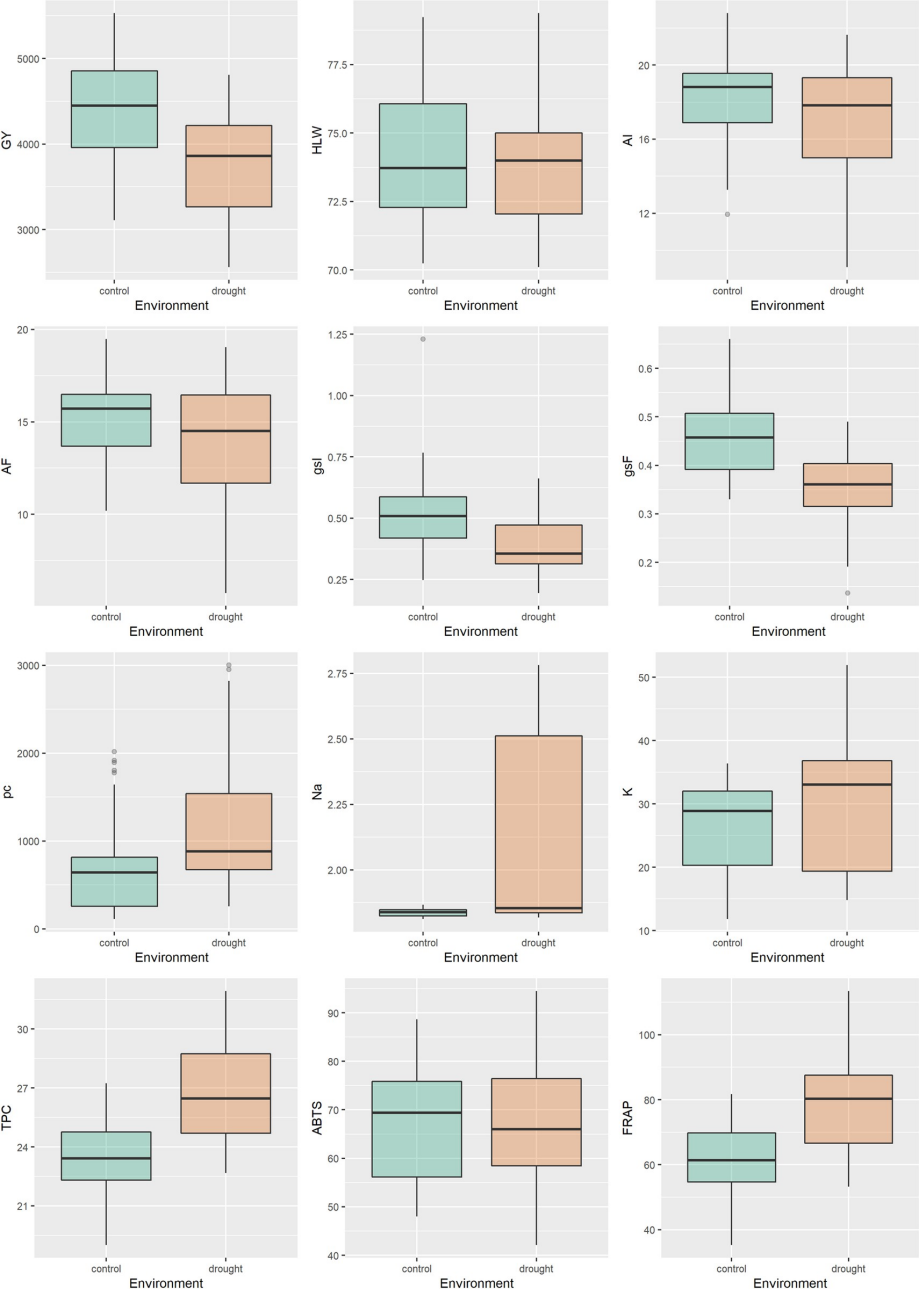
Results for box-plot of variables measured in 18 wheat genotypes under the two conditions of control and drought. Grain yield (GY, kg ha^-1^), hectolitre weight (HLW, kg hL^−1^), initial and final liquid photosynthesis (AI and AF μmol de CO_2_ m^- 2^ s^-1^), initial and final stomatal conductance (gsI and gsF mol H_2_O m^-2^ s^-1^), proline content (pc, μg g^-1^), sodium (Na) and potassium (K) (Na and K, mg g^-1^) total phenolic compounds (TPC, mg GAE g^-1^) and antioxidant activity by ABTS (mM TEAC g^-1^) and FRAP (mM Fe^+2^ g^-1^) methods.

In the box-plot, the results for the variables are presented individually in both environments (Fig 1). Environment control highlighted for GY, gsI, AI, gsF and AF in relation to drought. However, the drought environment presented high mean for TPC, FRAP, pc, Na and K. The variables K, AI and AF presented high amplitude of variation. Concomitant to this, the values of genotypes for potassium and sodium were higher under drought conditions. In both evaluations (initial and final), the variables stomatal conductance and net photosynthesis presented great variability of genotype responses in the environments (Figs 1, S2 and S3).

In fact, several and distinct biochemical-physiological responses occur as a defense of wheat and other plants against water deficit conditions. These environmental conditions affect plant performances, regardless of the [26,27]. The variation response in the agronomic and biochemical-physiological traits between the genotypes in each environment is also observed in the S2 and S3 Figs.

In this study, the wheat genotypes under the control condition were more productive than the drought environment. In fact, the drought condition affected the performance of the genotypes, which becomes more evident when we analyze the variables phenolic compounds, ABTS, FRAP and proline content. These data were highlighted in the heatmap (S2 and S3 Figs) with the average values of the genotypes for each environment and for each trait.

Since one of the main objectives of sustainable agriculture is the selection of wheat genotypes with greater tolerance to drought, together with high productivity or at least maintenance without loss in yield [44], the compounds involved in tolerance and response to these conditions must be evaluated. In this sense, phenolic compounds, compounds with antioxidant potential [45], are constantly produced by plants in response to a stressful situation, due to the oxidative stress generated. There is a cascade of crosstalk reactions in which these substances are produced to allow cell detoxification, generated by the excessive production of free radicals, in addition to intercellular communication [17,45,46]. The increased intensity of these compounds when compared to the initial state (control environment), as well as the antioxidant potential, can be observed in the drought environment for FRAP and TPC, especially in the BRS_264, VI_14001, VI_14050 and VI_14426 genotypes (S3D and S3F Fig).

The action of these compounds with antioxidant potential is associated with their structure reactivity in the number and substitution of the phenolic group(s), the hydroxyl(s) and the aromatic ring(s). In cereals, phenolic acids bound to substances in the cell wall [47] are the main phenolic compounds observed.

Understanding the mechanisms and response of genotypes to drought stress is crucial for research as it guides the positioning of genotypes in environments more likely to suffer from climatic fluctuations in precipitation. Wheat genotypes revealed variability for agronomic, physiological and biochemical traits, as observed in the range of variation of the box-plot analysis (Fig 1).

### 3.2 Variance components and genetic parameters

Under drought conditions, changes can be observed in cells, such as cycle and division, membranes, cell wall architecture, metabolism, accumulation of osmotically active substances, osmolytes and osmoprotectors and carbohydrate metabolism [48]. During severe water restriction, plants require several physiological adjustments to deal with dehydration. Wheat genotypes that assimilate and adjust more quickly to photosynthetic machinery will likely have greater tolerance to water stress or fluctuations in precipitation. Therefore, it is very important to extend information about genetic variability in different response variables, as shown in Table 2.

The results of genetic parameters (Table 2) reveal a heritability range between 0.029 Na and 0.85 for pc. The accuracy of selection (Ac) indicates interesting results for the variables evaluated, with a range of 0.17 Na to 0.92 pc. Concerning the results of genotype-environment correlation (rge), similar performance was observed for genotypes in the control and drought for the variables: Na, K, pc, FRAP and ABTS, and less association for gsI, AI, AF, HLW and TPC. The genotypic coefficient of variation (CV_g_) presents the greatest genetic variation, mainly for pc, K and AF, and lower genetic variation for HLW, Na, AI and gsF.

How much residual coefficient of variation (CV_r_) can be detected by good experimental precision, where higher CV_r_ was gsI 26.16%) and less HLW. Besides, eight out of 12 traits had CV_r_ less than 10%. The CV_g_/CV_r_ ratio value above 1 reveals that genetic variation was more important and bigger than the environmental variation. It occurred for GY, TPC, ABTS, FRAP, pc and K.

The results of the genetic parameter heritability (h^2^) and accuracy (h) are important to quantify the nature of the variable. For example, TPC and pc provided high genetic contribution. On the other hand, the physiological parameters, such as gsF, gsI, AI and Na, presented high environmental variation. According to the classification of [49], the experimental precision ranged from moderate to high. In the analysis of another important measure of experimental precision, the residual coefficient of variation (CVr%) was of high to moderate precision for the studied variables. It must be pointed out that the genetic coefficient (CVg%) presented high magnitude, which is expected, since the genotypes showed variability behavior for the evaluated traits.

### 3.3 Genotypic values—BLUP

**Fig 2:**
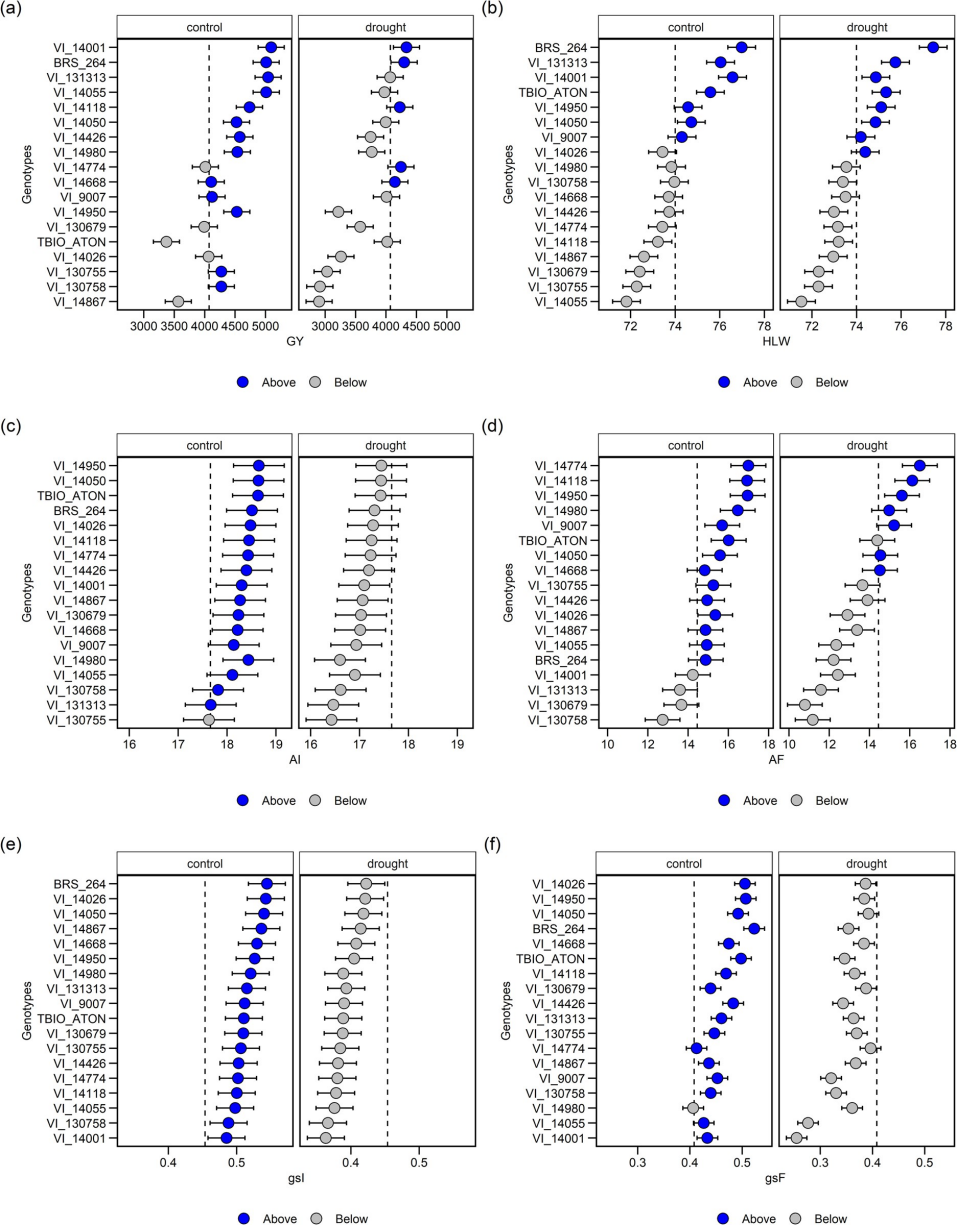
Results of BLUP values for 18 wheat genotypes evaluated under two conditions (control and stress) for variables grain yield (GY, kg ha^-1^) (a), hectolitre weight (HLW, kg hL^−1^) (b), rate photosynthetic liquid initial AI and final AF (AI and AF μmol de CO_2_ m^- 2^ s^-1^) (c and d), stomachal conductance initial gsI and final gsF (gsi and gsF, mol H_2_O m^-2^ s^-1^) (e and f).

In this study, 18 genotypes were evaluated under drought conditions. The grain yield (Fig 2A) of drought-stressed genotypes, except VI14774, VI14668, VI9007 and TBIO_ATON, was lower than that of the control plants, which corresponded to a reduction of 10.48 to 31.82% between the other genotypes, while the major reduction was found for VI_130758. The GY values were 3371.99 to 5093.67 for the control environment, and 2901.18 to 4337.03 kg ha-1 for drought environment. The genotypes VI_14001, BRS 264, VI_14118 and VI_14668 had higher GY for both environments, control and drought (Fig 2A), where the mean value genotypes were 4 ton ha^-1^. The strains VI_130755 and VI_130758 presented medium-high control and significant reduction for drought conditions. In the analysis of hectoliter weight (Fig 2B), the average HLW = 74 kg L stands out. It must be emphasized that no great impact of the environment was observed on this variable, where the same genotypes are above the average in both environments.

Drought tolerance is a complex phenomenon usually associated with a set of stresses that occur simultaneously, such as high temperature, excess radiation and low humidity [50]. The present research was a development of the field, where the plants were also exposed to these environmental conditions. The genotypes VI_14001, BRS_264, VI_14118 and VI_14668 presented adequate performance for the GY, for both environments.

A similar response occurred in the A and gs variables (Fig 2C–2F), and all the genotypes were negatively affected. The stomatal conductance was evaluated at two different times. First (initial), seven days after the beginning of the experiment, and for control (Fig 2E), all genotypes were with the BLUP mean of 0.4. Under stress, the values of the BLUP mean were below the general mean. A similar response can be observed with liquid photosynthesis in the initial evaluation (Fig 2C). In the evaluation at 25 days after the onset of drought stress (final measurement), the stomatal conductance continued (Fig 2E), with higher values in the control in relation to drought. And for liquid photosynthesis, in the second evaluation (Fig 2D), the genotypes in the control presented higher average than in drought.

**Fig 3:**
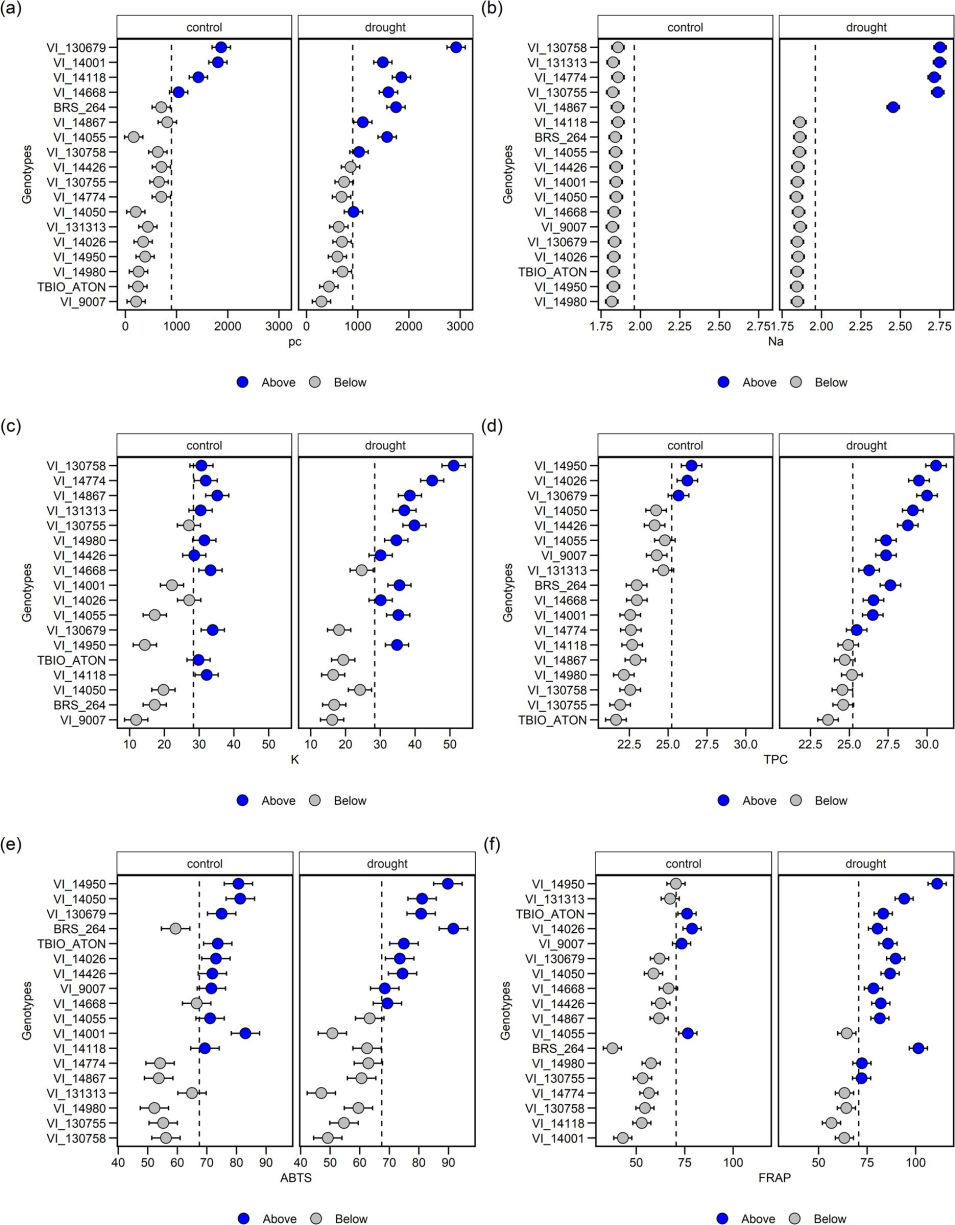
Results of BLUP values for 18 genotypes of wheat evaluated under two conditions (control and stress), for the variables proline content (pc, μg g^-1^) (a), sodium (Na, mg g^-1^) (b), potassium (K, mg g^-1^) (c), total phenolic compounds (TPC, mg GAE g^-1^) (d), antioxidant activity by ABTS (mM TEAC g^-1^) (e) and FRAP (mM Fe^+2^ g^-1^) (f).

Regarding pc (Fig 3A), the mean BLUP was close to 1000. It can be highlighted that 9 out of the 18 genotypes in the drought environment presented higher average values (VI_14050 strain). According to [51], the tendency to accumulate proline content is associated with a more immediate form of response as an indication of genotype tolerance, contrary to the more sensitive ones under drought conditions. It is, therefore, an important variable to be considered and evaluated in breeding studies. The drought stress generated an increase of up to 861.10% in pc (corresponding to 163.86 to 1574.86 μg g^-1^). The values for the 18 genotypes for this variable ranged from 163.86 to 1872.48 μg g^-1^ for control and from 292.53 to 2921.00 μg g^-1^ for the drought.

Low variation between genotypes was found for Na in the control environment (Fig 3B). However, in the drought environment, the VI_14867, VI_130755, VI_14774, VI_131313 and VI_130758 strains significantly increased the amount of sodium, above the BLUP mean. In this case, the drought led to a variation of up to 33% between genotypes, with values ranging from 1.84 to 2.75 mg g^-1^ (corresponding to VI_131313).

Most wheat genotypes increased the potassium concentration (Fig 3C) in the drought environment in relation to the control, and the mean BLUP was 29. VI_130758, VI_14774, VI_130755, VI_14950 VI_14055 and VI_14055 stood out under drought conditions. The highest levels observed were 51.09 and 44.92 mg g^-1^ for VI_130758 and VI_14774, respectively, under drought stress, while the lowest levels were obtained for VI_9007 (16.15 mg g^-1^), BRS_264 (16.75 mg g^-1^) and VI_4118 (16.46 mg g-1—presented a 48.83% reduction in the control for stress water).

Species differ greatly in their ability to circumvent water deficit. In sensitive species, physiological processes are affected by reduced tissue hydration. In tolerant species, their physiological and metabolic properties enable them to maintain a high level of tissue hydration even with a limited water supply [52]. Thus, both TPC production and antioxidant potential were significantly affected (Fig 3D).

In this study, under the drought condition, the genotypes increased the TPC mean values. Therefore, 12 out of the 18 genotypes are above the general average, mainly the strains VI_14950, VI_14026, VI_130679 and VI_14050. The values between genotypes ranged from 6.38% (VI_131313) to 20.35% (BRS_264). The three most responsive genotypes for this variable in descending order were BRS_264 (27.64 mg GAE g^-1^)> VI_14050 (30.58 mg GAE g^-1^)> VI_14426 (29.48 mg GAE g^-1^). However, the highest TPC value was found in the VI_14950 strain, with 30.84 mg GAE g^-1^, which corresponds to a 15.37% increase. The values found in the different genotypes under study agree with other studies that address water stress in wheat [53–55].

The antioxidant activity for the ABTS method presented greater variation between the control and drought environments for the genotypes BRS_264 54.3%> VI_14774 16.17%> VI14980 14.03%. In general, the ABTS values (Fig 3E) for the genotypes, regardless of the environment, ranged from 46.99 to 91.68 mM TEAC g^-1^. As complementary information, this variable revealed an average of 69 kg L^-1^, with the lines VI_14950, VI_14050 and cultivar BRS 264, with averages close to 80 in the drought environment.

In the evaluation of FRAP, the mean BLUP was 74 (Fig 3F), and 13 out of the 18 genotypes presented higher average in the dry environment in relation to the control. The genotypes BRS_264> VI_14026> VI_14050 presented the greatest variations between the control and drought environments, with values ​​from 56.62 mM Fe^+2^ g^-1^ (VI_14118) to 111.13 mM Fe^+2^ g^-1^ (VI_14950) under drought, and from 37.66 mM Fe^+2^ g^-1^ (BRS_264) to 78.77 mM Fe^+2^ g^-1^ in the control environment (VI_14026).

**Fig 4:**
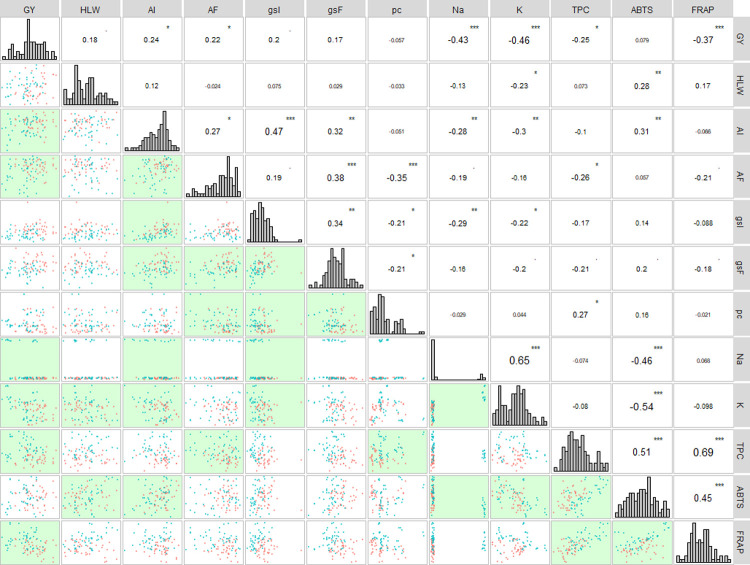
Pearson’s linear correlation between the ten studied traits. The lower diagonal shows the scatter plot where the environments are mapped with different point colors (Control = cyan and Drought = salmon). Grain yield (GY), hectolitre weight (HLW), initial liquid photosynthesis (AI) and final liquid photosynthesis (AF), initial stomatal conductance (gsI) and final stomatal conductance (gsF), proline content (pc), sodium (Na) and potassium (K), total phenolic compounds (TPC) and antioxidant activity by ABTS and FRAP methods.

According to these results, the antioxidant activity by ABTS exhibited a positive correlation with the total phenolic content (0.51) and the FRAP method (0.69) (Fig 4). These determinations are represented as stress indicators because they act as a defense of plants. Besides, several compounds belonging to this class are associated with many benefits previously reported in the literature, including antioxidant [56], anti-inflammatory [57], anti-cancer [58] and anti-microbial [59] activities, mainly in association with other nutritional and medicinal properties [60].

### 3.4 Multivariate and correlation analyses

#### 3.4.1. Principal components

**Fig 5:**
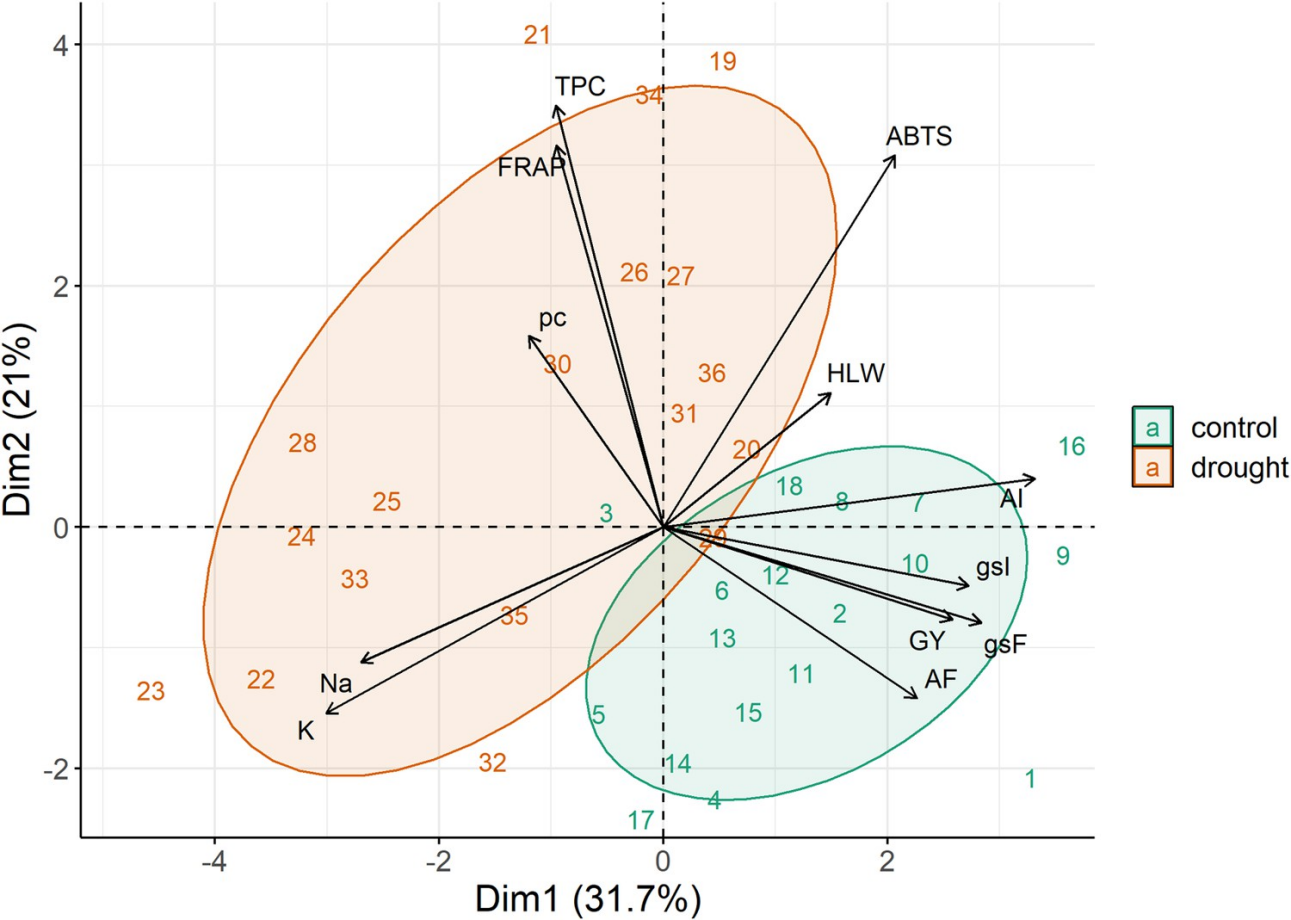
Principal component analysis for 18 wheat genotypes evaluated under two conditions (control and drought) 1 and 19-BRS 264, 2 and 20 -TBIO ATON, 3 and 21-VI 130679, 4 and 22-VI 130755, 5 and 23-VI 130758, 6 and 24-VI 131313, 7 and 25-VI 14001, 8 and 26-VI 14026, 9 and 27-VI 14050, 10 and 28-VI 14055, 11 and 29-VI 14118, 12 and 30-VI 14426, 13 and 31-VI 14668, 14 and 32-VI 14774, 15 and 33-VI 14867, 16 and 34-VI 14950, 17 and 35-VI 14980, 18 and 35-VI 9007 for 12 traits. Grain yield (GY), hectolitre weight (HLW), initial liquid photosynthesis (AI) and final liquid photosynthesis (AF), initial stomatal conductance (gsI) and final stomatal conductance (gsF), proline content (pc), total phenolic compounds (TPC) and antioxidant activity by ABTS and FRAP, sodium (Na) and potassium (K). Results of the contribution of variables for the environments jointed (b).

The results of the multivariate analysis via the main components (PCA) for the control and dry environments, considering 18 genotypes in each environment (Fig 5A), revealed that two PCAs explained 52.7% of the total variation. The variables AF, GY, gsF, gsI and AI were grouped in the control environment (green). On the other hand, the variables ABTS, TPC, FRAP, pc, Na and K are more associated with the drought (salmon) environment.

It is worth noting that the principal component analysis (Fig 5A) demonstrates the importance of analyzing the variables ABTS, TPC, FRAP, pc, Na and K, which is reinforced by the vip score (Fig 5B). A greater accumulation of these metabolites was expected, since, in a drought environment, plants accumulate protective compounds in an attempt to find cell homeostasis. A function of osmotic adjustment is observed for ions [13,24,25]. Results of PCA analysis are in accordance with linear correlations (Fig 4), where the main significance of negative correlations with 1 and 5% were between GY×Na (-0.43), GY×K (-0.46 GY×FRAP (-0.37) and GY×TPC (-0.25), in addition to ABTS×Na (-0.46), ABTS×K (-0.54), pc×AF (-0.35). On the other hand, the main positive significant correlations were found between GY×AF (0.22), GY×AI (0.24) and HLW×ABTS (0.28).

It is important to emphasize that, in addition to plants of different species presenting the likely accumulation of different substances, the same variability can occur between different genotypes [13]. Such fact, verified throughout the results of this study, led to the accumulation of different metabolites, consequently inducing the generation of various signaling cascades and response modulation. It is important to highlight that reduced GY is also associated with plants with low capacity to produce compounds of the plant defense system [44]. This feature can also be observed throughout this study.

#### 3.4.2. The importance of traits

In the analysis of the importance of the 12 variables evaluated in the 18 wheat genotypes for the environments together, the variables ABTS, TPC, K, AI and FRAP revealed greater contribution to the total variation (Fig 5B). Regarding the significance of the variables in the control condition, FRAP, K, ABTS, HLW, gsF and GY demonstrated greater contribution to total variation of wheat genotypes. In the drought environment, the variables FRAP, ABTS, K and gsI revealed greater contribution to the total variation. In other words, they are the variables that most contribute to explain the variability of wheat genotypes.

#### 3.4.3. Index selection

[61] performed the screening of wheat accessions for drought tolerance, and selected the best accessions using the MGIDI selection index, which allowed the identification of accessions with tolerance to the drought environment. Our selection results are presented below. The results of the selection gain analysis (Table 3) via multi-trait genotype ideotype analysis showed gains in the desired sense in eight of the 12 characters, which highlights a selection differential (DS) of 23.83% for pc. It is also important to mention that there was 4.07% of DS for GY.

**Table 3:** Original value (Xo), Selected value (Xs), Selection differential (SD) and Selection Differential in percentage (SD%) for the MGIDI in 18 wheat genotypes. Fig 6 of the manuscript presents the complete trait description.

Traits	Factor	Xo	Xs	SD	SD(%)	sense	goal
TPC	FA1	25.223	25.425	0.201	0.797	increase	100
ABTS	FA1	67.431	68.172	0.741	1.098	increase	100
K	FA1	28.376	28.363	-0.013	-0.045	increase	0
Na	FA1	1.960	1.957	-0.003	-0.173	decrease	100
AI	FA1	17.651	17.664	0.012	0.070	increase	100
FRAP	FA2	70.589	69.539	-1.049	-1.486	increase	0
gsI	FA2	0.453	0.442	-0.010	-2.326	decrease	100
gsF	FA2	0.407	0.400	-0.006	-1.704	decrease	100
pc	FA3	902.279	1117.310	215.031	23.831	increase	100
AF	FA3	14.451	14.148	-0.303	-2.100	increase	0
GY	FA4	4067.728	4233.464	165.736	4.074	increase	100
HLW	FA4	73.999	73.626	-0.372	-0.503	increase	0

Grain yield (GY), hectolitre weight (HLW), initial liquid photosynthesis (AI) and final liquid photosynthesis (AF), initial stomatal conductance (gsI) and final stomatal conductance (gsF), proline content (pc), total phenolic compounds (TPC) and antioxidant activity by ABTS and FRAP, sodium (Na) and potassium (K).

**Fig 6:**
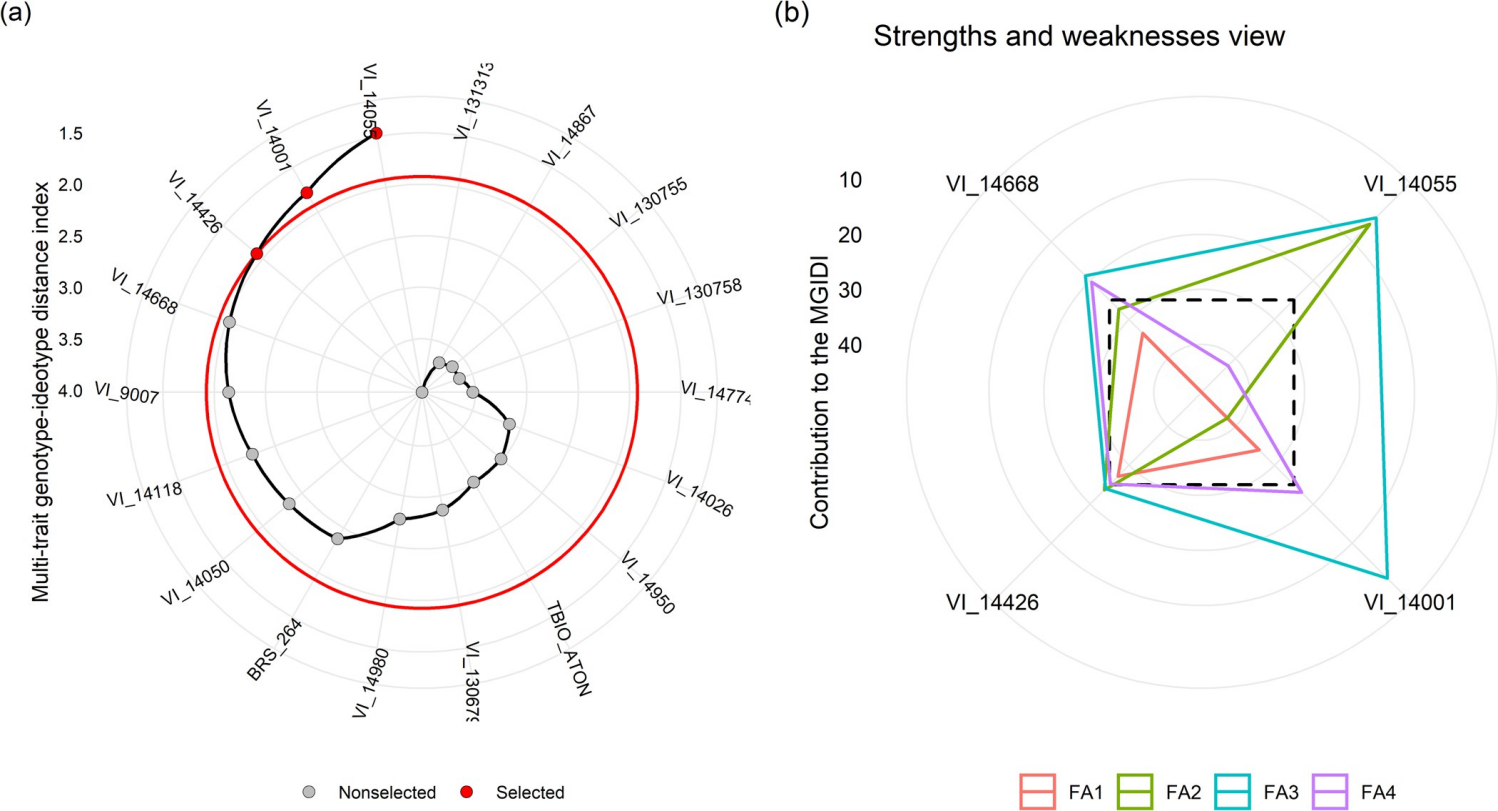
(a) Genotype ranking based on the multi-trait index. Selected genotypes are highlighted in red. (b) The strengths and weaknesses of genotypes are presented as the proportion of each factor on the computed multi-trait genotype-ideotype (MGIDI) of all genotypes. The smaller the proportion explained by a factor (closer to the external edge), the closer the traits within that factor to the ideotype. FA1: TPC (total phenolic compounds), ABTS, K (potassium), Na (sodium) and AI (initial liquid photosynthesis); FA2: FRAP, gsI (initial stomatal conductance) and gsF (final); FA3: pc (proline content) and AF (final); and FA4: GY (grain yield) and HLW (hectolitre weight).

The wheat genotypes were selected by the multivariate index, by which we simultaneously selected for control and drought. Out of the 18 genotypes studied, four strains were selected: VI_14055, VI_14001, VI_14426 and VI_1466 (Fig 6A), whose performance was similar to that of the ideotype used in MGIDI. On the other hand, the wheat lines of VI_131313 and VI_14867 presented higher sensitivity, since their performance was far from the desired.

In the analysis of the strengths and weaknesses of the four selected lines (Fig 6B and S2 Table), we observed the VI_14055 lineage with strength points to the factor 1 - (FA1: TPC, ABTS, K, Na and AI), and weak of VI_14426. For the factor 2 - (FA2: FRAP, gsI and gsF) VI_14001 lineage presented strong and weak point of VI_14055. To the factor 3 - (FA3: pc and AF) the VI_14426 and VI_14668 lineages presented strong points, and VI_14001 revealed weak point, and in the last factor 4 - (FA4: GY and HLW) the VI_14055 lineage presented strong point in this factor.

The results (Fig 6B) showed that the mechanisms of response to drought stress in the selected lines are distinct, thus several biochemical-physiological responses occur as a defense of wheat in water deficit conditions [26,27]. It is important to emphasize, to between different genotypes occur the accumulation antioxidant substances, where also occurred variations in the amount and profile of compounds, according to the condition and genotype involved [13,18]. It can occur also in reason of genetic variability of lineages, once that were generated of crosses different.

Lineage VI_14055 showed increased of TPC, ABTS e K in drought condition, these compounds are related to the antioxidant defense in wheat [15,16]. In this sense, phenolic compounds, compounds with antioxidant potential [45], are constantly produced by plants in response to the stress, due to the oxidative stress generated. Lineage VI_14001, showed as biochemical-physiological responses the stomatic conductance stability and FRAP inhibition and both environments. Lineages VI_14426 and VI_14668 showed increase the pc concentration and stomatic conductance in the drought condition (S2 Fig). The accumulation of osmoprotectors, such as proline, helps wheat to perform basic metabolic functions and mainly improves the maintenance of osmotic balance, the protection of organelles and cells facing dehydration, stabilization of membranes and structures of proteins and enzymes, and detoxification of ROS [24,25]. Tendency to accumulate proline content is associated with a more immediate form of response as an indication of genotype tolerance these genotypes evaluated. Multivariate selection indices are powerful tools for identifying genotypes with desirable performance, as they consider all variables of interest simultaneously. In our drought tolerance screening of the set of wheat strains, we were able to select three strains (VI_14055, VI_14001 and VI_14426) with desirable performance, based on agronomic, biochemical and physiological assessments.

## 5. Conclusion

The results obtained in this study suggest that the effect of drought stress can be exerted through a mechanism dependent on the wheat genotype involved. Lineages VI_14055, VI_14001 and VI_14426 stood out as the main potential for commercial use under conditions of water stress, due to their adequate agronomic, biochemical and physiological performance. Furthermore, it corroborates that the multi-trait genotype-ideotype distance index (MGIDI) is a very important tool and facilitator in data analysis for the faster and more efficient selection of strains.

## Supporting information

S1 Fig Results of the soil moisture gradient in the environments for two sampled depths, where 0th corresponds to the onset of drought stress and the 34th the end of stress. Viçosa-MG. UFV- Brazil. 2021.
(DOCX)

S2 Fig Results means of 18 genotypes wheat evaluated in the conditions drought and control to grain yield Grain yield (GY, kg ha^-1^), hectolitre weight (HLW, kg hL^−1^), rate photosynthetic initial and final (AI) (AI and AF μmol de CO_2_ m^- 2^ s^-1^) and estomatic conductance initial and final (gsI and gsF, mol H_2_O m^-2^ s^-1^). Viçosa–MG/Brazil 2021.
(DOCX)

S3 Fig Results means of 18 genotypes wheat evaluated in the conditions drought and control to proline content (pc, μg g^-1^), sodium (Na) (Na and K, mg g^-1^), potassium (K), total phenolic compounds (TPC, mg GAE g^-1^) and antioxidant activity by ABTS (mM TEAC g^-1^) and FRAP (mM Fe^+2^ g^-1^). Viçosa–MG/Brazil 2021.
(DOCX)

S1 Table Hypothesis testing for the fixed effects of environment (Env) and blocks within environments (E:Rep) for 12 traits available in 18 genotypes wheat in two environments. Viçosa–MG/Brazil, 2021.
(DOCX)

S2 Table Results of factor loadings of 18 wheat genotypes evaluated in control and drought environments. Viçosa-MG/Brazil 2021.
(DOCX)

S1 Graphical abstract (TIF)

S1 Data (XLSX)

## References

[1] Hossain A, Skalicky M, Brestic M, Maitra S, Ashraful AM; Syed MA, et al. Consequences and Mitigation Strategies of Abiotic Stresses in Wheat (*Triticum aestivum* L.) under the Changing Climate. Agron. 2021; 11: 241. Available in: 10.3390/agronomy11020241.

[2] Hazard B, Trafford K, Lovegrove A. et al. Strategies to improve wheat for human health. Nat Food. 2020; 1: 475–480. Available in: 10.1038/s43016-020-0134-6. 37128081

[3] Biel W., Kazimierska K., Bashutska U. (2020). Nutritional value of wheat, triticale, barley and oat grains. Acta Sci. Pol. Zootechnica, 19(2), 19–28. doi: 10.21005/asp.2020.19.2.03

[4] FAOSTAT Crops (Food and Agriculture Organization of the United Nations). 2021; Available in: https://www.fao.org/worldfoodsituation/csdb/en/.

[5] Shahbandeh M. Leading 10 wheat producers worldwide from 2016/2017 to 2020/21. 2021; Available in: https://www.statista.com/statistics/237908/global-top-wheat-producing-countries/.

[6] World Agricultural Production. World Wheat Production 2020/2021. 2021; Available in: http://www.worldagriculturalproduction.com/crops/wheat.aspx.

[7] FAO. 2018. The future of food and agriculture–Alternative pathways to 2050. Rome. 224 pp. Licence: CC BY-NC-SA 3.0 IGO.

[8] Beres BL, Hatfield JL, Kirkegaard JA, Eigenbrode SD, Pan WL, Lollato RP, et al. Toward a Better Understanding of Genotype × Environment × Management Interactions-A Global Wheat Initiative Agronomic Research Strategy. 2020. Front. Plant Sci. 2020; 11: 828. doi: 10.3389/fpls.2020.00828 32612624 PMC7308648

[9] Nezhadahmadi AP, Md Z, Faruq G. Drought Tolerance in Wheat. Sci. 2013; 610721. doi: 10.1155/2013/610721 24319376 PMC3844267

[10] Wani SH, Tripathi P, Zaid A. et al. Regulação transcricional da tolerância ao estresse osmótico em trigo (*Triticum aestivum* L.). Plant Mol Biol. 2018; 97, 469–487. doi: 10.1007/s11103-018-0761-6 30109563

[11] Yang D, Liu Y, Cheng H. et al. Genetic dissection of flag leaf morphology in wheat (*Triticum aestivum* L.) under diverse water regimes. BMC Genet. 2016; 17: 94. Available in: doi: 10.1186/s12863-016-0399-9 27352616 PMC4924301

[12] Baskar V, Venkatesh R, Ramalingam S. Flavonoids (Antioxidants Systems) in Higher Plants and Their Response to Stresses. In: Gupta D., Palma J., Corpas F. (eds) Antioxidants and Antioxidant Enzymes in Higher Plants. 2018. Springer, Cham. Available in: 10.1007/978-3-319-75088-0_12.

[13] Ahmad P, Jaleel C, Salem M, Nabi G, Sharma S. Roles of Enzymatic and non-enzymatic antioxidants in plants during abiotic stress. Crit. Rev. Biotechnol. 2010; 30: 161–75. doi: 10.3109/07388550903524243 20214435

[14] Chaudhry S, Sidhu GPS. Climate change regulated abiotic stress mechanisms in plants: a comprehensive review. Plant Cell Rep. 2021; Available in: doi: 10.1007/s00299-021-02759-5 34351488

[15] ShamLoo M, Babawale EA, Furtado A. et al. Effects of genotype and temperature on accumulation of plant secondary metabolites in Canadian and Australian wheat grown under controlled environments. Sci Rep. 2017; 7: 9133. Available in: doi: 10.1038/s41598-017-09681-5 28831148 PMC5567368

[16] Ma D, Sun D, Wang C, Li Y, Guo T. Expression of flavonoid biosynthesis genes and accumulation of flavonoid in wheat leaves in response to drought stress. Plant Physiol Biochem. 2014; 80:60–6. doi: 10.1016/j.plaphy.2014.03.024 Epub 2014 Mar 31. . 24727789

[17] Caverzan A, Casassola A, Brammer SP. Antioxidant responses of wheat plants under stress. Genet Mol Biol. 2016; 1:1–6. doi: 10.1590/1678-4685-GMB-2015-0109 27007891 PMC4807390

[18] Zhu D, Gengrui Z, Zhen Z, Zhimin W, Xing Y, Yueming Y. Effects of Independent and Combined Water-Deficit and High-Nitrogen Treatments on Flag Leaf Proteomes during Wheat Grain Development. Int. J. Mol. Sci. 2020; 21, 6: 2098. doi: 10.3390/ijms21062098 32204325 PMC7139553

[19] Kaur H PK Salh B Singh. Role of defense enzymes and phenolics in resistance of wheat crop (*Triticum aestivum* L.) towards aphid complex. J. Plant Interact. 2017; 12: 1, 304–311, doi: 10.1080/17429145.2017.1353653

[20] Ahanger MA, Qin C, Begum N. et al. Nitrogen availability prevents oxidative effects of salinity on wheat growth and photosynthesis by up-regulating the antioxidants and osmolytes metabolism, and secondary metabolite accumulation. BMC Plant Biol. 2019; 19: 479. doi: 10.1186/s12870-019-2085-3 31703619 PMC6839093

[21] Santos MCB, da Silva L, Luciana R, Nascimento FR, do Nascimento TP, Cameron LC, et al. Metabolomic approach for characterization of phenolic compounds in different wheat genotypes during grain development. Int. Food Res. 2018; S0963996918306409–. doi: 10.1016/j.foodres.2018.08.034 31466630

[22] Chen Z, Ma Y, Weng Y, Yang R, Gu Z, Wang P. Effects of UV-B radiation on phenolic accumulation, antioxidant activity and physiological changes in wheat (Triticum aestivum L.) seedlings. Food Biosci. 2019; 30: 100409–. doi: 10.1016/j.fbio.2019.04.010 30902306

[23] Lou L, Li X, Chen J, Li Y, Tang Y, Lv J. Photosynthetic and ascorbate-glutathione metabolism in the flag leaves as compared to spikes under drought stress of winter wheat (Triticum aestivum L.). PLoS ONE. 2018; 13 (3): e0194625. Available in: doi: 10.1371/journal.pone.0194625 29566049 PMC5864061

[24] Hasan Humna. Climate Change and Food Security with Emphasis on Wheat || Role of osmoprotectants and drought tolerance in wheat. 2020; 207–216. Available in: doi: 10.1016/B978-0-12-819527-7.00013–3

[25] Nadeem M. Climate Change and Food Security with Emphasis on Wheat || Role of osmoprotectants in salinity tolerance inÂ wheat. 2020; 93–106. Available in: doi: 10.1016/B978-0-12-819527-7.00006–6

[26] Aslam M, Maqbool MA, Cengiz R. Mechanisms of Drought Resistance. In: Drought Stress in Maize (Zea mays L.) (pp.19–36). 2015. Cham. Available in: 10.1007/978-3-319-25442-5_3.

[27] Ali M, Gul A, Hasan H, Gul S, Fareed A, Nadeem M, Siddique R, Jan SU, Jamil M. Cellular mechanisms of drought tolerance in wheat. 2020. 10.1016/B978-0-12-819527-7.00009–1. Available in: doi: 10.1016/b978-0-12-819527-7.00009–1

[28] Olivoto T, Nardino M. MGIDI: toward an effective multivariate selection in biological experiments. Bioinformatics. 2021 Jun 16;37(10):1383–1389. doi: 10.1093/bioinformatics/btaa981 . 33226063

[29] ZADOKS J. c., CHANG T. T.; KONZAK C. F. A decimal code for the growth stages ofcereals. Weed Research, Oxford, v. 14, p. 415–421,1974.

[30] Bates L.S., Waldren R.P. & Teare I.D. Rapid determination of free proline for water-stress studies. Plant Soil 39, 205–207 (1973). 10.1007/BF00018060.

[31] Habib N, Ali Q, Ali S, Javed MT, Zulqurnain HM, Perveen R, et al. Use of Nitric Oxide and Hydrogen Peroxide for Better Yield of Wheat (Triticum aestivum L.) under Water Deficit Conditions: Growth, Osmoregulation, and Antioxidative Defense Mechanism. Plants. 2020; 9(2): 285–. doi: 10.3390/plants9020285(2020) 32098385 PMC7076392

[32] Zhang H, Kim MS, Sun Y, Dowd SE, Shi H, Paré PW. Soil Bacteria Confer Plant Salt Tolerance by Tissue-Specific Regulation of the Sodium TransporterHKT1. Mol. Plant Microbe Interact. 2008; 21, 6: 737–744. 18624638 10.1094/MPMI-21-6-0737

[33] Zhang J-L, Aziz M, Qiao Y, Han Q-Q, Li J, Wang Y-Q, et al. Soil microbe Bacillus subtilis (GB03) induces biomass accumulation and salt tolerance with lower sodium accumulation in wheat. Crop Pasture Sci. 2014; 65: 423.

[34] Singh R, Rathore D. Oxidative stress defence responses of wheat (Triticum aestivum L.) and chilli (Capsicum annum L.) cultivars grown under textile effluent fertilization. Plant Physiol. Biochem. 2018; 123: 342–358. doi: 10.1016/j.plaphy.2017.12.027 29294440

[35] Singleton VL, Orthofer R, Lamuela-Ravento´s RM. Analysis of total phenols and other oxidation substrates and antioxidants by means of Folin-Ciocalteu reagent. Method Enzymol. 1999; 299: 152–178.

[36] Re R, Pellegrini N, Proteggente A, Pannala A, Yang M, Rice-Evans C. Antioxidant activity applying an improved ABTS+ radical cation decolorization assay. Free Radical Bio. Med. 1999; 26: 1231–1237. Available in: doi: 10.1016/s0891-5849(98)00315-3 10381194

[37] Pulido R, Bravo L, Saura-Calixto F. Antioxidant activity of dietary polyphenols as determined by a modified ferric reducing/ antioxidant power assay. J. Agric. Food Chem. 2000; 48: 3396–3402. Available in: doi: 10.1021/jf9913458 10956123

[38] Olivoto T, Nardino M. MGIDI: towards an effective multivariate selection in biological experiments. 2020; doi: 10.1590/0001-3765202020180874 33226063

[39] Wei T, Simko V (2021). R package ’corrplot’: Visualization of a Correlation Matrix. (Version 0.90), https://github.com/taiyun/corrplot.

[40] Wheeler T, von Braun J. Climate Change Impacts on Global Food Security. Science (New York, N.Y.). 2013; 341: 508–13. doi: 10.1126/science.1239402 23908229

[41] Ray DK, Mueller ND, West PC, Foley JA. Yield Trends Are Insufficient to Double Global Crop Production by 2050. PLoS ONE. 2013; 8(6): e66428. doi: 10.1371/journal.pone.0066428 23840465 PMC3686737

[42] Curtis T, Halford NG. Food security: the challenge of increasing wheat yield and the importance of not compromising food safety. Ann Appl Biol. 2014; 164 (3): 354–372. doi: 10.1111/aab.12108 Epub 2014 Feb 21. ; PMCID: PMC4240735. 25540461 PMC4240735

[43] Crossa J, Pérez-Rodríguez P, Cuevas J, Montesinos-López O, Jarquín D, de Los Campos G, et al. Genomic Selection in Plant Breeding: Methods, Models, and Perspectives. Trends Plant Sci. 2017; 11: 961–975. doi: 10.1016/j.tplants.2017.08.011 Epub 2017 Sep 28. . 28965742

[44] Kirova E, Pecheva D, Simova L. Drought response in winter wheat: protection from oxidative stress and mutagenesis effect. Acta Physiol. Plant. 2021; 43. doi: 10.1007/s11738-021-03334-x 34776557 PMC8578917

[45] Rahman M, Akond M, Babar M, Beecher C, Erickson J, Thomason K, et al. LC-HRMS Based Non-Targeted Metabolomic Profiling of Wheat (Triticum aestivum L.) under Post-Anthesis Drought Stress. Am. J. Plant Sci. 2017; 8: 3024–3061. doi: 10.4236/ajps.2017.812205

[46] Afridi MS, Amna S, Mahmood T, Salam A, Mukhtar T, Mahmood S, et al. Induction of tolerance to salinity in wheat genotypes by plant growth promoting endophytes: Involvement of ACC deaminase and antioxidant enzymes. Plant Physiol. Biochem. 2019; 139: 569–577. doi: 10.1016/j.plaphy.2019.03.041 31029030

[47] ZILIC S.; KOCADAĞLI T.; VANČETOVIĆ J.; GÖKMEN V. Effects of baking conditions and dough formulations on phenolic compound stability, antioxidant capacity and color of cookies made from anthocyanin-rich corn flour.LWT—Food Science and Technology, 2016, v.65, p. 597–603.

[48] Bi H, Kovalchuk N, Langridge P, Tricker P, Lopato S, Borisjuk N. The impact of drought on wheat leaf cuticle properties. BMC Plant Biol. 2017; 17: 85. doi: 10.1186/s12870-017-1033-3 28482800 PMC5422891

[49] Resende MDV, Duarte JB. Precisão e controle de qualidade em experimentos de avaliação de cultivares. PAT. 2007; S. l, 37, 3: 182–194, 2007. Available in: https://www.revistas.ufg.br/pat/article/view/1867.

[50] Kurian M, Ardakanian R, Veiga LG, Meyer K. Resources, services and risks: how can data observatories bridge the science-policy divide in environmental governance? ISBN 978-3-319–28706–5 (eBook). Switzerland: Springer, 2016. Available in: https://link.springer.com/book/10.1007/978-3-319-28706-5.

[51] Saeedipour S. Relationship of grain yield, ABA and proline accumulation in tolerant and sensitive wheat cultivars as affected by water stress. Proc. Natl. Acad. Sci.U.S.A. 2013; 83: 311–315. doi: 10.1007/s40011-012-0147-5

[52] Fritsche-Neto R, Borém A. Plant Breeding for Abiotic Stress Tolerance. 2012; Available in: SBN 978-3-642-30553-5 (eBook) doi: 10.1007/978-3-642-30553-5

[53] Islam MZ, Park BJ, Lee YT. Effect of salinity stress on bioactive compounds and antioxidant activity of wheat microgreen extract under organic cultivation conditions. Int J Biol Macromol. 2019; 1,140: 631–636. doi: 10.1016/j.ijbiomac.2019.08.090 Epub 2019 Aug 12. . 31415860

[54] Kiani R, Arzani A, Mirmohammady Maibody SAM. Polyphenols, Flavonoids, and Antioxidant Activity Involved in Salt Tolerance in Wheat, Aegilops cylindrica and Their Amphidiploids. Front Plant Sci. 2021; 25;12: 646221. doi: 10.3389/fpls.2021.646221 33841475 PMC8027307

[55] Tomé-Sánchez I, Martín-Diana AB, Peñas E, Bautista-Expósito S, Frias J, Rico D, et al. Soluble Phenolic Composition Tailored by Germination Conditions Accompany Antioxidant and Anti-Inflammatory Properties of Wheat. Antioxidants 2020; 9: 426. doi: 10.3390/antiox9050426 32423164 PMC7278661

[56] Fogarasi AL, Kun S, Tankó G, Stefanovits-Bányai E, Hegyesné-Vecseri B. A comparative assessment of antioxidant properties, total phenolic content of einkorn, wheat, barley and their malts. Food Chem. 2015; 15: 167:1–6. doi: 10.1016/j.foodchem.2014.06.084 Epub 2014 Jul 1. . 25148951

[57] Whent M, Huang H, Xie Z, Lutterodt H, Yu L, Fuerst EP, et al. Phytochemical composition, anti-inflammatory, and antiproliferative activity of whole wheat flour. J Agric Food Chem. 2012; 7, 60(9):2129–35. doi: 10.1021/jf203807w Epub 2012 Feb 22. . 22321109

[58] Porrang S, Rahemi N, Davaran S, Mahdavi M, Hassanzadeh B. Preparation and in-vitro evaluation of mesoporous biogenic silica nanoparticles obtained from rice and wheat husk as a biocompatible carrier for anti-cancer drug delivery. Eur J Pharm Sci. 2021; 1;163:105866. doi: 10.1016/j.ejps.2021.105866 Epub 2021 May 4. . 33957220

[59] Saha S, Islam Z, Islam S, Hossain Md, Islam SM. Evaluation of antimicrobial activity of wheat (*Triticum aestivum* L.) against four bacterial strains. 2018; 20. 58–62.

[60] Pawan Yadav. Nutritional Contents and Medicinal Properties of Wheat: A Review. Life sci. med. res. 2011; LMSR-22.

[61] Pour-Aboughadareh A, Poczai P. Dataset on the use of MGIDI index in screening drought-tolerant wild wheat accessions at the early growth stage. Data in Brief. 2021; Available in: doi: 10.1016/j.dib.2021.107096 34307802 PMC8257987

