# Supplementary material for: Understanding drought response mechanisms in wheat and multi-trait selection
Source: PLoS One. 2022 Apr 14;17(4):e0266368. doi: 10.1371/journal.pone.0266368 (PMC9009675; doi:10.1371/journal.pone.0266368)
Supplement: S1 Fig — Viçosa-MG. UFV- Brazil. 2021. (DOCX) [file pone.0266368.s001.docx]

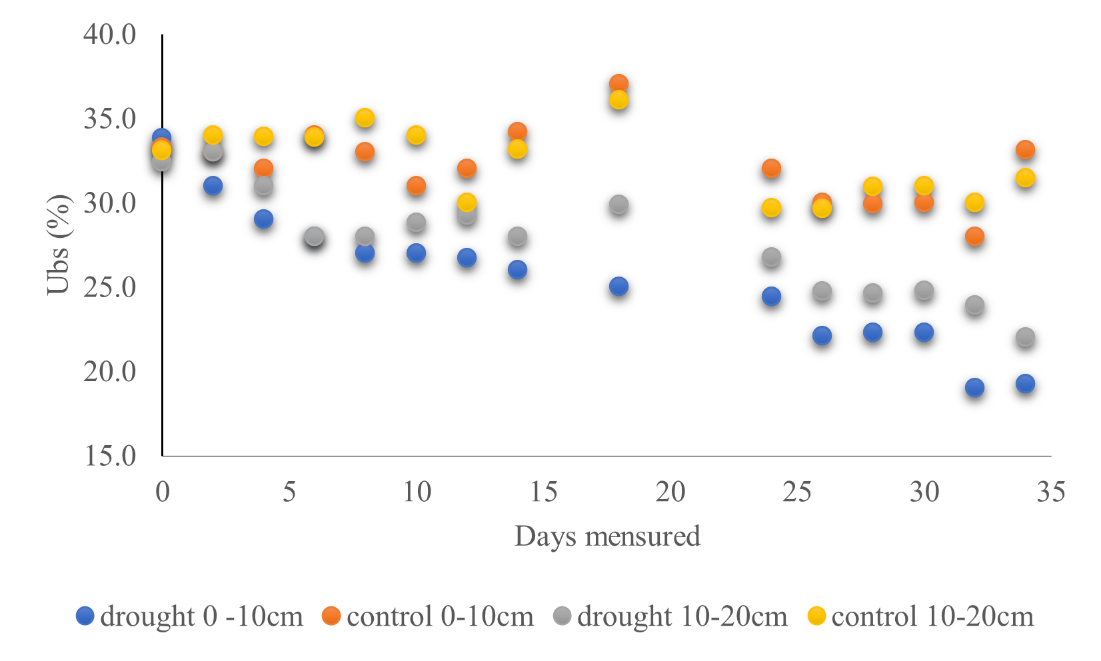


Supporting information S1- Results of the soil moisture gradient in the environments for two sampled depths, where 0th corresponds to the onset of drought stress and the 34th the end of stress. Viçosa-MG. UFV- Brazil. 2021.
