# Supplementary material for: Understanding drought response mechanisms in wheat and multi-trait selection
Source: PLoS One. 2022 Apr 14;17(4):e0266368. doi: 10.1371/journal.pone.0266368 (PMC9009675; doi:10.1371/journal.pone.0266368)
Supplement: S2 Fig — Viçosa–MG/Brazil 2021. (DOCX) [file pone.0266368.s002.docx]

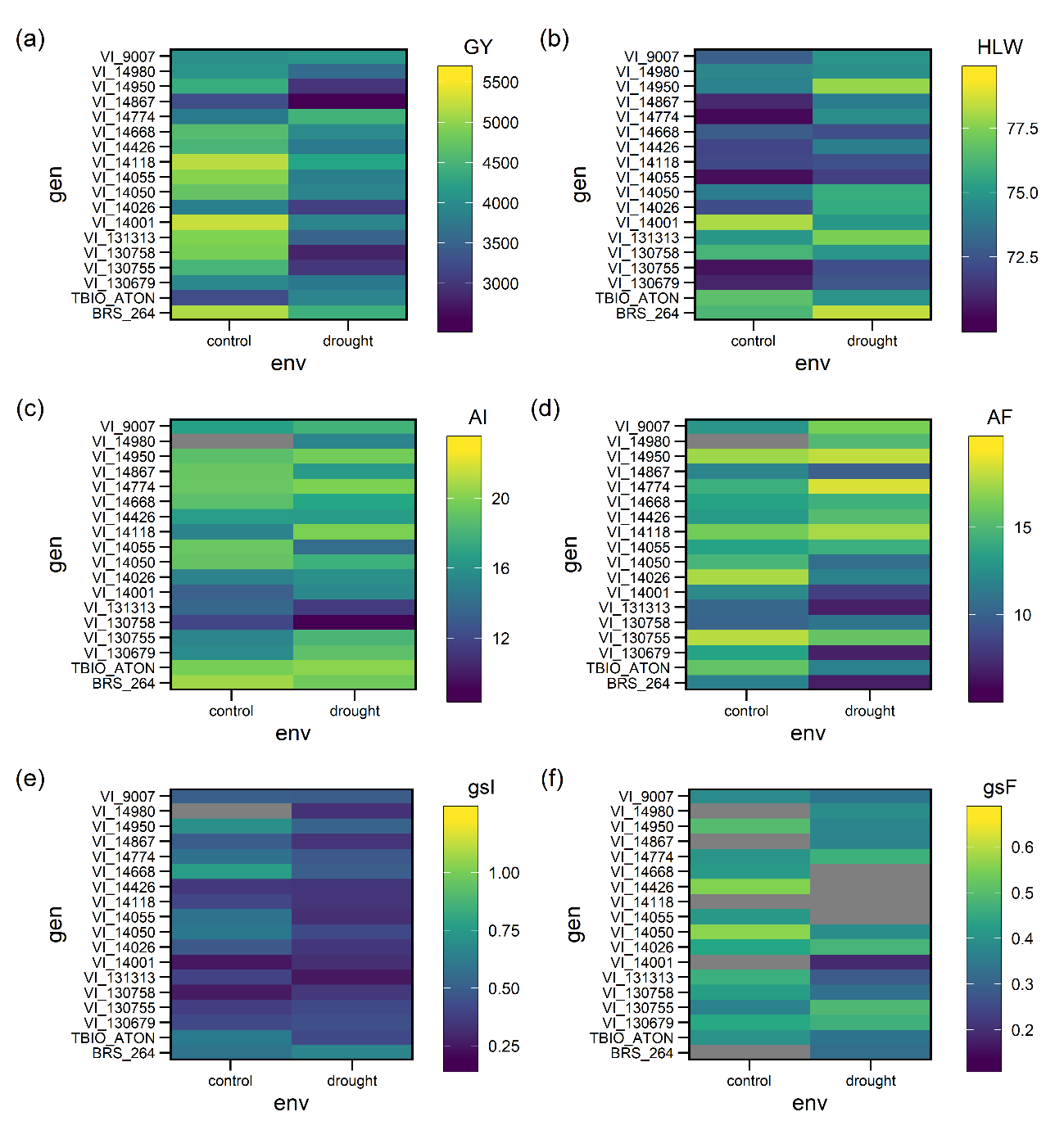


Supporting Information S3 **-** Results means of 18 genotypes wheat evaluated in the conditions drought and control to grain yield Grain yield (GY, kg ha^-1^), hectolitre weight (HLW, kg hL^−1^), rate photosynthetic initial and final (AI) (AI and AF μmol de CO_2_ m^- 2^ s^-1^) and estomatic conductance initial and final (gsI and gsF, mol H_2_O m^-2^ s^-1^). Viçosa – MG/Brazil 2021.
