# Supplementary material for: Understanding drought response mechanisms in wheat and multi-trait selection
Source: PLoS One. 2022 Apr 14;17(4):e0266368. doi: 10.1371/journal.pone.0266368 (PMC9009675; doi:10.1371/journal.pone.0266368)
Supplement: S3 Fig — Viçosa–MG/Brazil 2021. (DOCX) [file pone.0266368.s003.docx]

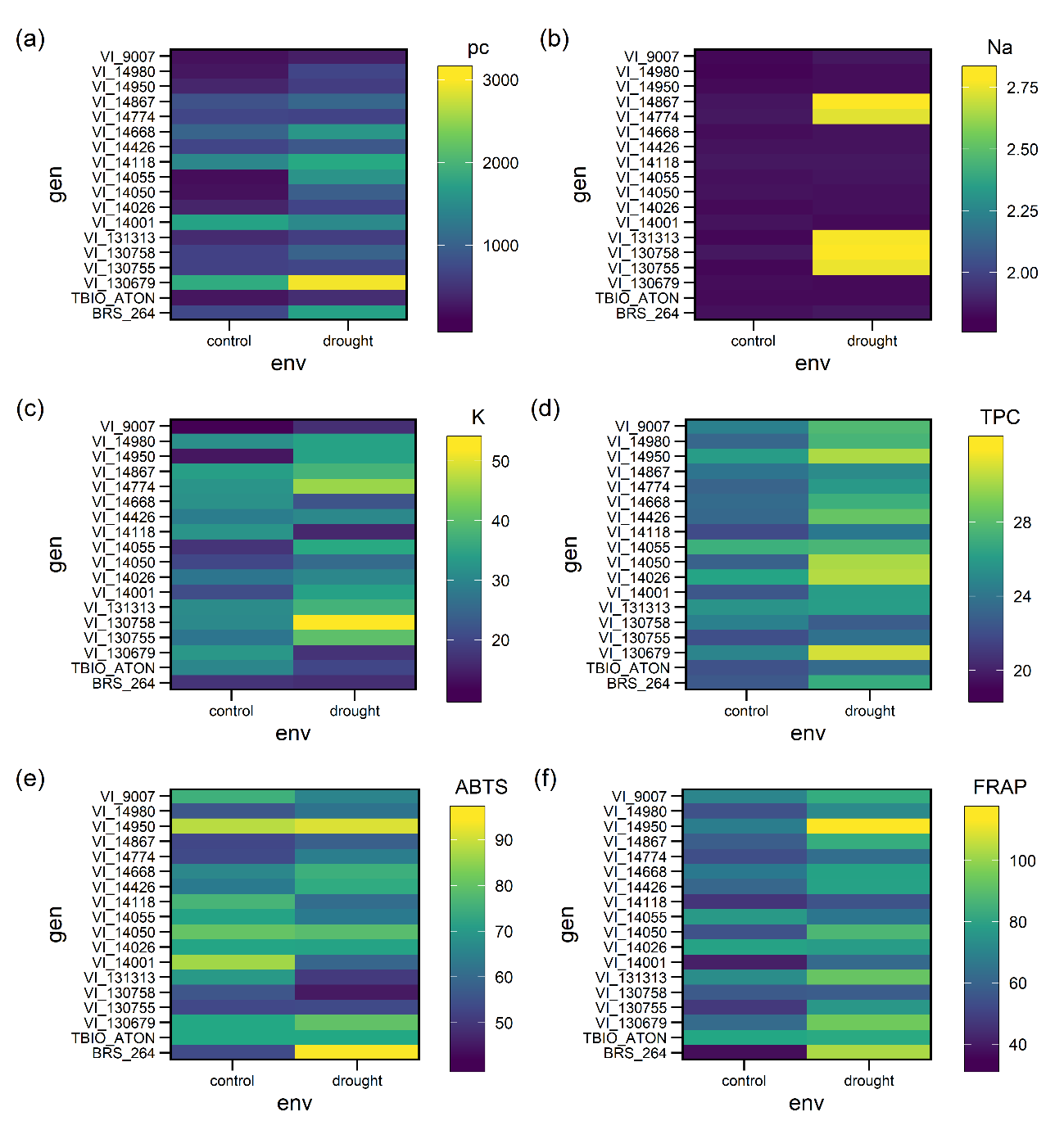
Supporting Information S4 - Results means of 18 genotypes wheat evaluated in the conditions drought and control to proline content (pc, µg g^-1^), sodium (Na) (Na and K, mg g^-1^), potassium (K), total phenolic compounds (TPC, mg GAE g^-1^) and antioxidant activity by ABTS (mM TEAC g^-1^) and FRAP (mM Fe^+2^ g^-1^). Viçosa – MG/Brazil 2021.
