# Supplementary material for: Understanding drought response mechanisms in wheat and multi-trait selection
Source: PLoS One. 2022 Apr 14;17(4):e0266368. doi: 10.1371/journal.pone.0266368 (PMC9009675; doi:10.1371/journal.pone.0266368)
Supplement: S1 Table — Viçosa–MG/Brazil, 2021. (DOCX) [file pone.0266368.s004.docx]

Supporting Information S2 - Hypothesis testing for the fixed effects of environment (Env) and blocks within environments (E:Rep) for 12 traits available in 18 genotypes wheat in two environments. Viçosa – MG/Brazil, 2021.

| Traits | Source | SumSq | Mean Sq | NumDF | DenDF | F value | Pr(>F) |
| --- | --- | --- | --- | --- | --- | --- | --- |
| GY | E | 867210.55 | 867210.55 | 1 | 28.74 | 9.53 | 0.0044 |
| GY | E/Rep | 408580.53 | 102145.13 | 4 | 67.14 | 1.12 | 0.3530 |
| HLW | E | 0.65 | 0.65 | 1 | 62.35 | 0.28 | 0.5938 |
| HLW | E/Rep | 36.86 | 9.21 | 4 | 68.00 | 4.04 | 0.0053 |
| AI | E | 0.01 | 0.01 | 1 | 83.75 | 0.00 | 0.9621 |
| AI | E/Rep | 142.43 | 35.60 | 4 | 83.96 | 6.46 | 0.0001 |
| AF | E | 18.33 | 18.33 | 1 | 63.87 | 3.28 | 0.0748 |
| AF | E/Rep | 58.96 | 14.74 | 4 | 64.19 | 2.63 | 0.0418 |
| gsI | E | 0.21 | 0.21 | 1 | 84.21 | 15.12 | 0.0002 |
| gsI | E/Rep | 0.24 | 0.06 | 4 | 84.41 | 4.40 | 0.0028 |
| gsF | E | 0.09 | 0.09 | 1 | 49.86 | 21.28 | 0.0000 |
| gsF | E/Rep | 0.02 | 0.01 | 4 | 52.30 | 1.45 | 0.2295 |
| pc | E | 42460.16 | 42460.16 | 1 | 17.69 | 15.10 | 0.0011 |
| pc | E/Rep | 8924.17 | 2231.04 | 4 | 68.00 | 0.79 | 0.5333 |
| Na | E | 0.04 | 0.03 | 1 | 19.36 | 4.68 | 0.0431 |
| Na | E/Rep | 0.03 | 0.01 | 4 | 68.00 | 1.07 | 0.3748 |
| K | E | 2.46 | 2.46 | 1 | 17.43 | 1.98 | 0.1762 |
| K | E/Rep | 1.22 | 0.30 | 4 | 68.00 | 0.24 | 0.9107 |
| TPC | E | 69.52 | 69.52 | 1 | 43.74 | 50.32 | 0.0000 |
| TPC | E/Rep | 7.45 | 1.86 | 4 | 68.00 | 1.34 | 0.2609 |
| ABTS | E | 0.49 | 0.49 | 1 | 21.68 | 0.02 | 0.8738 |
| ABTS | E/Rep | 45.49 | 11.37 | 4 | 68.00 | 0.59 | 0.6698 |
| FRAP | E | 314.65 | 314.65 | 1 | 19.38 | 20.85 | 0.0002 |
| FRAP | E/Rep | 125.11 | 31.27 | 4 | 68.00 | 2.07 | 0.0939 |

Grain yield (GY), hectolitre weight (HLW), liquid photosynthesis initial (AI) and final (AF), stomatal conductance initial (gsI) and final (gsF), proline content (pc), total phenolic compounds (TPC) and antioxidant activity by ABTS and FRAP, sodium (Na) and potassium (K).
