# Supplementary material for: Understanding drought response mechanisms in wheat and multi-trait selection
Source: PLoS One. 2022 Apr 14;17(4):e0266368. doi: 10.1371/journal.pone.0266368 (PMC9009675; doi:10.1371/journal.pone.0266368)
Supplement: S2 Table — Viçosa-MG/Brazil 2021. (DOCX) [file pone.0266368.s005.docx]

Supporting Information S5 – Results of factor loadings of 18 wheat genotypes evaluated in control and drought environments. Viçosa-MG/Brazil 2021.

| Genotype | FA1 | FA2 | FA3 | FA4 |
| --- | --- | --- | --- | --- |
| BRS_264 | 22.071 | 31.771 | 7.746 | 38.410 |
| TBIO_ATON | 29.273 | 25.627 | 43.928 | 1.170 |
| VI_130679 | 7.118 | 18.689 | 31.539 | 42.653 |
| VI_130755 | 50.107 | 10.877 | 17.006 | 22.007 |
| VI_130758 | 70.108 | 9.294 | 1.584 | 19.011 |
| VI_131313 | 50.641 | 24.677 | 5.865 | 18.815 |
| **VI_14001** | **33.923** | **42.092** | **0.937** | **23.046** |
| VI_14026 | 24.260 | 45.524 | 10.915 | 19.300 |
| VI_14050 | 19.545 | 46.364 | 33.851 | 0.238 |
| **VI_14055** | **48.712** | **5.506** | **3.854** | **41.925** |
| VI_14118 | 13.868 | 17.301 | 46.735 | 22.094 |
| **VI_14426** | **27.164** | **23.656** | **23.972** | **25.206** |
| **VI_14668** | **33.497** | **27.352** | **18.753** | **20.396** |
| VI_14774 | 38.128 | 2.748 | 40.902 | 18.220 |
| VI_14867 | 37.945 | 21.204 | 14.072 | 26.778 |
| VI_14950 | 11.690 | 47.567 | 26.468 | 14.273 |
| VI_14980 | 52.493 | 3.912 | 36.126 | 7.468 |
| VI_9007 | 32.722 | 27.805 | 36.509 | 2.962 |
