# Supplementary figures and images for: Understanding drought response mechanisms in wheat and multi-trait selection
Source: PLoS One. 2022 Apr 14;17(4):e0266368. doi: 10.1371/journal.pone.0266368 (PMC9009675; doi:10.1371/journal.pone.0266368)

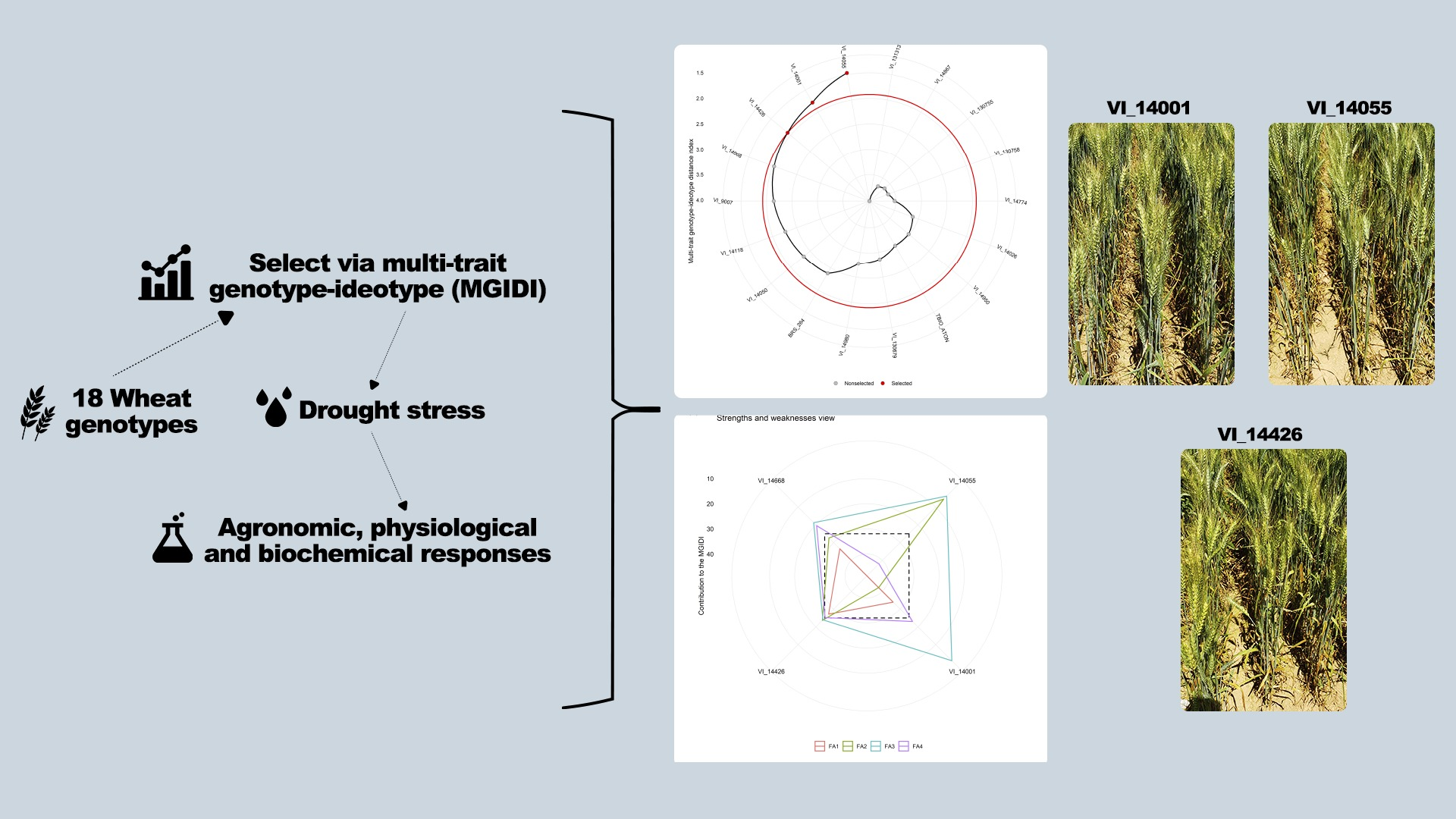

Supplement: S1 Graphical abstract — (TIF) [file pone.0266368.s006.tif]
